# GAIT SPEED AT THE ACUTE PHASE PREDICTED HEALTH-RELATED QUALITY OF LIFE AT 3 AND 12 MONTHS AFTER STROKE: A PROSPECTIVE COHORT STUDY

**DOI:** 10.2340/jrm.v56.24102

**Published:** 2024-04-15

**Authors:** Yishuang ZHAO, Xiaoling LIAO, Hongqiu GU, Yong JIANG, Yingyu JIANG, Yongjun WANG, Yumei ZHANG

**Affiliations:** 1 Department of Rehabilitation Medicine; 2 Department of Neurology, Beijing Tiantan Hospital, Capital Medical University, Beijing; 3 China National Clinical Research Center for Neurological Diseases, Beijing; 4 National Center for Healthcare Quality Management in Neurological Diseases, Beijing; 5 Advanced Innovation Center for Human Brain Protection, Capital Medical University, Beijing, China

**Keywords:** health-related quality of life, gait speed, stroke, EQ-5D-3L

## Abstract

**Objective:**

To investigate the association between acute-phase gait speed and health-related quality of life (HRQoL) at 3 and 12 months post-stroke.

**Design:**

Prospective cohort study.

**Subjects/Patients:**

1,475 patients with first-ever ischaemic stroke.

**Methods:**

The patients were divided into 3 groups according to tertiles of gait speed, namely ≤0.8, 0.8–1.1, ≥1.1 m/s. Gait speed was assessed by the 10-m walking test within 2 weeks of hospitalization for acute stroke and before the rehabilitation programme. HRQoL measurements include the 3-level EuroQol five dimensions (EQ-5D-3L) index and EuroQoL visual analogue scale (EQ-VAS) scores. Linear and logistic regression analyses were used to identify associations between gait speed and HRQoL.

**Results:**

Adjusted for all covariates, the highest gait speed tertile group were associated with higher EQ-5D-3L index (B = 0.0303 and B = 0.0228, respectively, *p* < 0.001), and higher EQ-VAS (B = 3.3038 and B = 3.8877, respectively, *p* < 0.001), and lower odds of having problems with mobility (OR = 2.55 [95% CI: 0.141–0.458] and 0.485 [0.289–0.812], respectively, *p* < 0.01), self-care (OR = 0.328 [95% CI: 0.167–0.646] and 0.412 [0.217–0.784], respectively, *p* < 0.01), and usual activities (OR = 0.353 [95% CI: 0.211–0.590] and 0.325 [0.198–0.536], respectively, *p* < 0.0001) at 3 and 12 months, and pain/discomfort at 12 months (OR = 0.558 [95% CI:0.335–0.930], *p* < 0.05).

**Conclusion:**

Acute-phase gait speed was predictive of post-stroke HRQoL at 3 and 12 months, especially when associated with domain-specific EQ-5D-3L.

LAY ABSTRACT

Walking ability is one of the main priorities of persons who have suffered a stroke and has a large impact on health-related quality of life. The 10-m walking test at a comfortable gait speed has been used extensively in measuring walking ability in the acute phase of stroke. Other studies have shown that stroke patients perceive lower health-related quality of life than the general population in the first years after stroke. Therefore, this study aimed to investigate the association between acute-phase gait speed and post-stroke health-related quality of life at 3 and 12 months. This study indicated that stroke patients with acute-phase low speed had worse health-related quality of life and higher risk of having problems with mobility, self-care, and usual activities at 3 and 12 months after stroke, and increased risk of pain/discomfort problems at 12 months. In terms of clinical implications, an initial focus on patients’ walking abilities may have a significant impact on health-related quality of life.

Cumulative evidence has consistently demonstrated that stroke patients have a lower health-related quality of life (HRQoL) than that of healthy individuals (1, 2). Previous studies have identified the impact of different factors on HRQoL after stroke, including demographic factors (age, female sex, and low education level) and clinical factors (stroke severity, stroke type, smoking, and depression) (3–5). However, data are scarce on the impact of gait speed at admission on stroke patients’ future quality of life.

Gait speed, measured as the time required to walk a short distance at a comfortable pace, is one of the most common methods for assessing gait ability that can be easily and quickly evaluated in the laboratory as well as at the clinical site (6). Indeed, gait speed has been regarded as the sixth vital sign and assessed clinically along with breathing, temperature, heart rate, pain, and blood pressure (7). Grau-Pellicer et al. (8) have demonstrated that improved gait speed after a multimodal rehabilitation programme was associated with community mobility and quality of life in patients with chronic stroke. Furthermore, a previous cross-sectional study showed significant correlations between gait speed and physical domains of quality of life, and community ambulators, whose gait speed was more than 0.8 m/s, had a higher quality of life than that of household or limited community ambulators (9). However, previous studies have evaluated gait speed in the chronic phase after stroke, but not in the acute phase after stroke (8, 9). The acute phase of stroke is a critical time for enhanced neural plasticity and spontaneous neurological recovery and should be a target for recovery trials (10). The 10-m walking test (10MWT) is a relevant measurement tool in the acute phase (11). However, inadequate data exist on gait speed in the acute phase of stroke with longitudinal HRQoL.

Therefore, this study aimed to investigate the association between acute-phase gait speed and HRQoL at 3 and 12 months of follow-up in patients with ischaemic stroke. We hypothesized that a slower gait speed at admission would be associated with a worse HRQoL after 3 and 12 months.

## METHODS

### Study design and participants

The detailed design, rationale, and basic description of the Third China National Stroke Registry-III (CNSR-III) have been previously described in detail (12). Briefly, CNSR-III is a multicentre, prospective, cohort study aimed at studying vascular risk factors, clinical characteristics, diagnosis, treatment, and prevention for patients with acute stroke. This study enrolled consecutive patients with stroke from CNSR-III between August 2015 and March 2018. The inclusion criteria for participation were as follows: age >18 years; diagnosis of first-ever ischaemic stroke within 7 days; completion of the 10MWT at baseline; ability to communicate and stable clinical conditions in both levels of consciousness and vital signs. Patients were excluded if they had severe aphasia, hearing loss, visual impairment, difficulty cooperating, severe unilateral neglect, or dyslexia. Individuals whose baseline 10MWT results were not available and patients who had not completed the assessment of HRQoL at 3 and 12 months were excluded.

According to the Helsinki Declaration, this study was approved by the Ethics Committees of Beijing Tiantan Hospital (No. KY2015-001-01) and all participating centres (12). Written informed consent for inclusion was signed by patients or legally authorized representatives.

### Baseline data collection

Data collected at baseline included sociodemographic information (age, sex, education, body mass index, living status, marital status, income, health insurance, occupation class, current smoking, and current drinking), medical histories (hypertension, diabetes, hyperlipidaemia, atrial fibrillation, coronary artery disease, heart failure, peripheral arterial disease, arthritis), intravenous thrombolysis, rehabilitation status. Physical activity was defined according to the classification of this variable in the CNSR-III database. Briefly, participants’ self-reported levels of physical activity were measured by the duration and perceived intensity of weekly participation in certain physical activities, including farm work, work, housework, transport-related physical activity, leisure exercise, or sports. Physical activity patterns were defined as follows: None, no physical activity; Low, not reporting any moderate- or vigorous-intensity physical activities; Medium, reporting less than 4 h/week of moderate-intensity physical activity and less than 80 min/week of vigorous-intensity physical activity; High, reporting at least 4h/week of moderate-intensity physical activity or at least 80 min/week of vigorous-intensity physical activity. Stroke severity was assessed with the National Institutes of Health Stroke Scale (NIHSS) on a scale ranging from 0 to 42, with a lower value indicating lesser stroke severity (13). The degree of functional disability was assessed with the modified Rankin Scale (mRS) (score 0–5) (14). The Montreal Cognitive Assessment Scale (MoCA) is a screening tool for detecting mild cognitive impairment. The score ranges from 0 to 30 where a value below 26 can indicate cognitive impairment (15).

Gait speed was assessed by the 10MWT within 2 weeks of hospitalization for acute stroke and before the rehabilitation programme. Standardized instructions and a demonstration were provided before each walking test. Calculating a comfortable gait speed involved measuring the time (in seconds) needed to travel the middle 10 m of a 14-m walkway. A walking aid, crutches, and foot orthoses were permitted in this test and to be supported by one personal assistant in the event of instability or balance problems. If walking aids or support were required, they were provided at retest to ensure that testing procedures were equivalent. Calculating a comfortable gait speed involved measuring the time (in seconds) needed to travel the middle 10 m of a 14-m walkway. The timing starts when the subject’s first foot crosses the 2-m mark and stops when the first foot crosses the 12-m mark. The test was provided without any motivation (16). The test was repeated 3 times. The average of the 3 walking tests was recorded as the gait speed. The 10MWT demonstrated excellent inter-rater reliability, test–retest reliability, construct validity, and sensitivity to change in people with acute stroke (16, 17).

### Outcomes and follow-up

The main clinical outcome was HRQoL after 3 and 12 months of follow-up. EuroQol five dimensions (EQ-5D), developed by the EuroQol Group, was used to measure HRQoL (18), which included a health state descriptive system with 5 dimensions and a visual analogue scale (VAS) score.

The 3-level EuroQol five dimensions (EQ-5D-3L) scale includes 5 dimensions (i.e., mobility, self-care, usual activities, pain/discomfort, and anxiety/depression) are each rated on 3 levels (i.e., G1 = no problems, G2 = some problems, and G3 = severe problems) (19). The EuroQoL visual analogue scale (EQ-VAS) is a visual scale with vertical isometric scales used to evaluate the overall health status of individuals, ranging from the best imaginable health (100) to the worst imaginable health (0) (20). The EQ-5D-3L has also been validated for the measurement of HRQoL in patients with stroke (19). The EQ-VAS scores can be obtained directly from the questionnaire, while the EQ-5D-3L index needs to be converted into health utility values when describing the quality of life. Therefore, we adopted the time trade-off (TTO) method based on the Chinese population to calculate the health utility values (21). The calculation formula for health utility score is as follows: Utility = 1 − (constant + sum of all coefficients × variable values). Specifically, when calculating the utility based on the value set of China 2014 (21):

Utility = 1 − (0.039 + 0.099 × M2 + 0.246 × M3 + 0.105 × S2 + 0.208 × S3 + 0.074 × U2 + 0.193 × U3 + 0.092 × P2 + 0.236 × P3 + 0.086 × A2 + 0.205 × A3 + 0.022 × N3).

In the formula, M2, S2, U2, P2, and A2 respectively represent mobility, self-care, usual activities, pain/discomfort, and depression/anxiety at level 2, and the value is 1 and 0 for the others. M3, S3, U3, P3, and A3 indicate that when the above dimensions are at level 3, the value is 1, and 0 otherwise. N3 is equal to 1 if at least 1 of the 5 dimensions is at level 3, and 0 otherwise. We reported the values of the EQ-VAS score and EQ-5D-3L index in each group, as well as the frequencies of each level in EQ-5D dimension.

### Statistical analysis

The data analysis was conducted with SAS 9.4 (SAS Institute Inc, Cary, NC, USA). Continuous variables are expressed as the means with standard deviation (SD) or median with interquartile range (IQR), as appropriate. Categorical data are presented as proportions. Patients were divided into 3 groups according to the tertiles of gait speed, with Q1 as the lowest tertile and Q3 as the highest tertile. Baseline characteristics were analysed by χ^2^ statistics or Fisher’s exact test for the categorical variables and 1-way ANOVA or Kruskal‒Wallis test for the continuous variables, as appropriate.

Multivariate linear regression analysis was used to determine the associations between gait speed and HRQoL. Multivariate logistic regression analysis was used to evaluate the associations of gait speed with the 5 dimensions of the EQ-5D-3L, using odds ratios (ORs) and 95% confidence intervals (CIs). Baseline variables that were considered clinically relevant or that showed a univariate relationship with outcome were entered into a multivariate regression model. Variables for inclusion were carefully chosen, given the number of events available, to ensure the parsimony of the final model. Then, 3 models were constructed. Model 1 was adjusted for age, sex, educational level, household income, health insurance, and occupation class; Model 2 was further adjusted for medical history (hypertension, diabetes mellitus, hyperlipidaemia, and atrial fibrillation), current smoking status, and current drinking status; and Model 3 was further adjusted for physical activity, rehabilitation training status, and clinical scale scores (NIHSS, mRS, MoCA). Sensitivity analysis was performed by using the UK value sets (22). All *p*-values were two-sided, and a *p*-value < 0.05 was considered statistically significant.

## RESULTS

### Study participants and characteristics

**Fig. 1:**
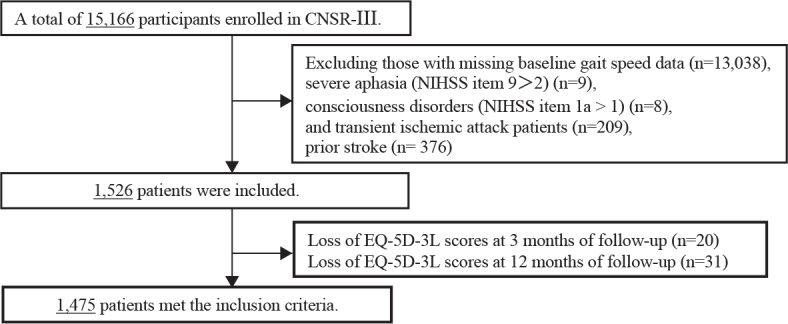
Flowchart. CNSR-III: the Third China National Stroke Registry-III; NIHSS: National Institutes of Health Stroke Scale; EQ-5D-3L: 3-level EuroQol five dimensions.

In total, 15,166 ischaemic stroke participants were ultimately eligible for inclusion and had complete information at baseline in the CNSR-III. However, we excluded 13,038 patients who had missing gait speed data at baseline, 376 prior stroke patients, and 209 transient ischaemic attack patients. Seventeen participants were excluded for severe aphasia and consciousness disorders, and 51 patients were excluded due to missing EQ-5D-3L scores at 3 and 12 months of follow-up. Therefore, the final cohort consisted of 1,475 patients (Fig. 1).

The baseline clinical and assessment characteristics of the cohort stratified by tertiles of gait speed are given in Table I. All enrolled patients were divided into 3 groups according to the tertile of gait speed: Q1, ≤0.80 m/s; Q2, 0.80–1.10 m/s; and Q3, ≥1.10 m/s. Significant differences in age, sex, educational levels, household income, health insurance, occupation class, smoking status, drinking status, history of hypertension, diabetes, hyperlipidaemia, coronary heart disease, rehabilitation training received, physical activity, baseline mRS score, baseline NIHSS score, and MoCA score differed among the groups with different gait speed levels (all *p* < 0.05).

**Table I:** Baseline characteristics of the enrolled participants according to the tertiles of gait speed

Characteristics	Total	Gait speed			*p*-value
		Q1 (≤0.80 m/s)	Q2 (0.80–1.10 m/s)	Q3 (≥1.10 m/s)	
Participants, *n* (%)	1,475	463 (33.0)	451 (30.9)	561 (36.1)	
Demographic factors					
Age (years), mean (SD)	59.5 (10.5)	62.3 (10.9)	59.9 (10.3)	56.9 (9.8)	< 0.0001
Sex, *n* (%)					< 0.0001
Male	1,093 (74.1)	308 (66.5)	331 (73.4)	454 (80.9)	
Female	382 (25.9)	155 (33.5)	120 (26.6)	107 (19.1)	
Educational level, *n* (%)					< 0.0001
Unknown	131 (8.9)	39 (8.4)	38 (8.4)	54 (9.6)	
Primary school or below	358 (24.3)	110 (23.8)	95 (21.1)	153 (27.3)	
Junior school	548 (37.2)	143 (30.9)	171 (37.9)	234 (41.7)	
High school	377 (25.6)	143 (30.9)	128 (28.4)	106 (18.9)	
College or above	61 (4.1)	28 (6.0)	19 (4.2)	14 (2.5)	
Living situation, *n* (%)					0.4370
With someone	78 (5.3)	29 (6.3)	24 (5.3)	25 (4.5)	
Alone	1,397 (94.7)	434 (93.7)	427 (94.7)	536 (95.5)	
Marital status, *n* (%)					0.3412
Unmarried	7 (0.5)	2 (0.4)	2 (0.4)	3 (0.5)	
Married	1,408 (95.5)	434 (93.7)	434 (96.2)	540 (96.3)	
Divorced or widowed	58 (3.9)	26 (5.6)	15 (3.3)	17 (3.0)	
Remarried	1 (0.1)	1 (0.2)	–	–	
Unknown	1 (0.1)	–	–	1 (0.2)	
Monthly per capita household income (RMB yuan), *n* (%)					0.0013
< 700	49 (3.3)	9 (1.9)	14 (3.1)	26 (4.6)	
700 ≤ income < 1,500	245 (16.6)	74 (16.0)	79 (17.5)	92 (16.4)	
1,500 ≤ income < 2,300	398 (27.0)	112 (24.2)	116 (25.7)	170 (30.3)	
> 2,300	532 (36.1)	172 (37.1)	153 (33.9)	207 (36.9)	
Unknown	251 (17.0)	96 (20.7)	89 (19.7)	66 (11.8)	
Types of health insurance, *n* (%)					0.0040
UEMIS	577 (39.1)	170 (36.7)	174 (38.6)	233 (41.5)	
URMIS	334 (22.6)	96 (20.7)	123 (27.3)	115 (20.5)	
NRMIS	487 (33.0)	161 (34.8)	135 (29.9)	191 (34.0)	
Commercial	4 (0.3)	2 (0.4)	2 (0.4)	–	
Self-payment	52 (3.5)	20 (4.3)	13 (2.9)	19 (3.4)	
Others	21 (1.4)	14 (3.0)	4 (0.9)	3 (0.5)	
Occupation class, *n* (%)					0.0010
Institutions and agencies	191 (12.9)	48 (10.4)	58 (12.9)	85 (15.2)	
Businessman	77 (5.2)	16 (3.5)	32 (7.1)	29 (5.2)	
Production staff	485 (32.9)	145 (31.3)	155 (34.4)	185 (33.0)	
Transport staff	63 (4.3)	19 (4.1)	18 (4.0)	26 (4.6)	
Retired	378 (25.6)	157 (33.9)	103 (22.8)	118 (21.0)	
No job	125 (8.5)	31 (6.7)	39 (8.6)	55 (9.8)	
Others	156 (10.6)	47 (10.2)	46 (10.2)	63 (11.2)	
Clinical factors					
BMI (kg/m^2^), mean (SD)	25.1 (3.2)	24.8 (3.3)	25.2 (3.0)	25.2 (3.2)	0.1026
Medical history, *n* (%)					
Hypertension	869 (58.9)	292 (63.1)	266 (59.0)	311 (55.4)	0.0473
Diabetes	312 (21.2)	116 (25.1)	96 (21.3)	100 (17.8)	0.0187
Hyperlipidaemia	126 (8.5)	59 (12.7)	29 (6.4)	38 (6.8)	0.0005
Atrial fibrillation	64 (4.3)	29 (6.3)	22 (4.9)	13 (2.3)	0.0068
Coronary heart disease	130 (8.8)	50 (10.8)	40 (8.9)	40 (7.1)	0.1194
Heart failure	4 (0.3)	2 (0.4)	2 (0.4)		0.0343
Peripheral arterial disease	3 (0.2)		1 (0.2)	2 (0.4)	0.4495
Arthritis	19 (1.3)	9 (1.9)	5 (1.1)	5 (0.9)	0.3050
Intravenous thrombolysis, *n* (%)	156 (10.6)	39 (8.4)	46 (10.2)	71 (12.7)	0.0862
Received rehabilitation training, *n* (%)					
Hospitalization	468 (31.7)	213 (46.0)	128 (28.4)	127 (22.6)	< 0.0001
3 months after discharge	90 (6.1)	50 (10.8)	21 (4.7)	19 (3.4)	< 0.0001
12 months after discharge	67 (4.5)	42 (9.1)	14 (3.1)	11 (2.0)	< 0.0001
Lifestyle characteristics					
Current smoking status, *n* (%)	574 (38.9)	145 (31.3)	169 (37.5)	260 (46.3)	< 0.0001
Current drinking status, *n* (%)	318 (21.6)	86 (18.6)	86 (19.1)	146 (26.0)	0.0047
Physical activity, *n* (%)					< 0.0001
None	518 (35.1)	201 (43.4)	146 (32.4)	171 (30.5)	
High	423 (28.7)	102 (22.0)	130 (28.8)	191 (34.0)	
Medium	521 (35.3)	152 (32.8)	171 (37.9)	198 (35.3)	
Low	13 (0.9)	8 (1.7)	4 (0.9)	1 (0.2)	
Baseline assessment					
NIHSS on admission, median (IQR)	1.0 (0.0–2.0)	1.0 (0.0–3.0)	1.0 (0.0–2.0)	1.0 (0.0–2.0)	< 0.0001
mRS at admission, median (IQR)	1.0 (1.0–2.0)	2.0 (1.0–3.0)	1.0 (1.0–2.0)	1.0 (1.0–2.0)	< 0.0001
MoCA score, mean (SD)	21.7 (5.6)	20.0 (6.2)	22.2 (5.1)	22.7 (5.1)	< 0.0001

SD: standard deviation; UEMIS: urban employee medical insurance; URMIS: urban residents’ medical insurance; NRMIS: new rural medical insurance; BMI: body mass index; NIHSS: the National Institutes of Health Stroke Scale; IQR: interquartile range; mRS: modified Rankin Scale; MoCA: Montreal Cognitive Assessment.

Table II indicates the EQ-5D-3L index, the weighted proportions of problems for each of the EQ-5D-3L dimensions, and the EQ-VAS scores according to gait speed tertile level at 3 and 12 months. As the gait speed levels increased, the EQ-5D-3L index and EQ-VAS scores significantly increased at both 3 and 12 months (all *p* < 0.0001; Table II). The proportion of people having no problems with mobility, self-care, usual activity, and pain/discomfort significantly tended to increase as the gait speed increased at 3 and 12 months (all *p* < 0.05; Table II). At 12 months, the proportions of people having no problems with depression/anxiety were higher among those with the highest gait speed tertile than among those with the lowest gait speed tertile (*p* < 0.05; Table II).

**Table II:** EQ-5D-3L scores at 3 and 12 months based on gait speed

Variable		Total	Gait speed			*p*-value
			Q1 (≤0.80 m/s)	Q2 (0.80–1.10 m/s)	Q3 (≥1.10 m/s)	
Participants, *n* (%)		1,475	463 (33.0)	451 (30.9)	561 (36.1)	
3 months						
EQ-5D-3L index, mean (SD)		0.91 (0.11)	0.88 (0.14)	0.92 (0.10)	0.94 (0.07)	< 0.0001
EQ-VAS, mean (SD)		83.20 (13.17)	79.94 (14.17)	83.13 (12.58)	85.94 (12.14)	< 0.0001
EQ-5D-3L dimensions, *n* (%)						
Mobility	G1	1,331 (90.2)	371 (80.1)	416 (92.2)	544 (97.0)	< 0.0001
	G2	135 (9.2)	86 (18.6)	32 (7.1)	17 (3.0)	
	G3	9 (0.6)	6 (1.3)	3 (0.7)	–	
Self-care	G1	1,373 (93.1)	394 (85.1)	431 (95.6)	548 (97.7)	< 0.0001
	G2	93 (6.3)	63 (13.6)	18 (4.0)	12 (2.1)	
	G3	9 (0.6)	6 (1.3)	2 (0.4)	1 (0.2)	
Usual activity	G1	1,301 (88.2)	362 (78.2)	403 (89.4)	536 (95.5)	< 0.0001
	G2	167 (11.3)	97 (21.0)	45 (10.0)	25 (4.5)	
	G3	7 (0.5)	4 (0.9)	3 (0.7)	–	
Pain/discomfort	G1	1,311 (88.9)	389 (84.0)	410 (90.9)	512 (91.3)	0.0011
	G2	160 (10.8)	71 (15.3)	40 (8.9)	49 (8.7)	
	G3	4 (0.3)	3 (0.6)	1 (0.2)	–	
Depression/anxiety	G1	1,309 (88.7)	398 (86.0)	397 (88.0)	514 (91.6)	0.0579
	G2	153 (10.4)	59 (12.7)	51 (11.3)	43 (7.7)	
	G3	13 (0.9)	6 (1.3)	3 (0.7)	4 (0.7)	
12 months						
EQ-5D-3L index, mean (SD)		0.91 (0.11)	0.88 (0.14)	0.91 (0.11)	0.93 (0.08)	< 0.0001
EQ-VAS, mean (SD)		83.46 (12.71)	80.67 (13.09)	82.65 (13.21)	86.43 (11.32)	< 0.0001
EQ-5D-3L dimensions, *n* (%)						
Mobility	G1	1,316 (89.2)	378 (81.6)	404 (89.6)	534 (95.2)	< 0.0001
	G2	141 (9.6)	79 (17.1)	42 (9.3)	20 (3.6)	
	G3	18 (1.2)	6 (1.3)	5 (1.1)	7 (1.2)	
Self-care	G1	1,375 (93.2)	404 (87.3)	426 (94.5)	545 (97.1)	< 0.0001
	G2	82 (5.6)	49 (10.6)	20 (4.4)	13 (2.3)	
	G3	18 (5.6)	10 (2.2)	5 (1.1)	3 (0.5)	
Usual activity	G1	1,297 (87.9)	366 (79.0)	398 (88.2)	533 (95.0)	< 0.0001
	G2	163 (11.1)	90 (19.4)	48 (10.6)	25 (4.5)	
	G3	15 (1.0)	7 (1.5)	5 (1.1)	3 (0.5)	
Pain/discomfort	G1	1,336 (90.6)	396 (85.5)	411 (91.1)	529 (94.3)	< 0.0001
	G2	132 (8.9)	62 (13.4)	40 (8.9)	30 (5.3)	
	G3	7 (0.5)	5 (1.1)	–	2 (0.4)	
Depression/anxiety	G1	1,329 (90.1)	404 (87.3)	405 (89.8)	520 (92.7)	0.0169
	G2	129 (8.7)	51 (11.0)	44 (9.8)	34 (6.1)	
	G3	17 (1.2)	8 (1.7)	2 (0.4)	7 (1.2)	

G1: no problems; G2: some problems; G3: severe problems; EQ-5D-3L: 3-level EuroQol five dimensions; SD: standard deviation; EQ-VAS: EQ visual analogue scale.

### Associations between gait speed levels and HRQoL

We subsequently analysed these associations by dividing the gait speed results into tertiles. The results of tertile-based multiple linear regression analyses are listed in Table III. Adjusted for all covariates, the highest gait speed tertile group had a significantly higher EQ-5D-3L index (B = 0.0303 [95% CI:0.0168–0.0437] and B = 0.0228 [95% CI:0.0079–0.0377, respectively, *p* < 0.01) and EQ-VAS scores (B = 3.3038 [95% CI:1.5753–5.0324] and B = 3.8877 [95% CI:2.2170–5.5583], respectively, *p* < 0.001) than those in the lowest gait speed tertile group at 3 and 12 months. In sensitivity analyses, we found that the results remained similar when the EQ-5D-3L index was calculated by using the UK value sets (Tables SI and SII).

**Table III:** Association between acute-phase gait speed and post-stroke HRQoL scores at 3 and 12 months

Outcomes	Unadjusted		Model 1		Model 2		Model 3	
	B (95% CI)	*p*-value	B (95% CI)	*p*-value	B (95% CI)	*p*-value	B (95% CI)	*p*-value
*3 months*								
EQ-5D-3L index								
Q1	Ref.		Ref.		Ref.		Ref.	
Q2	0.0431 (0.0299–0.0564)	< 0.0001	0.0397 (0.0263–0.0530)	< 0.0001	0.0396 (0.0262–0.0530)	< 0.0001	0.0208 (0.0074–0.0341)	0.0024
Q3	0.0606 (0.0480–0.0732)	< 0.0001	0.0539 (0.0408–0.0670)	< 0.0001	0.0535 (0.0404–0.0667)	< 0.0001	0.0303 (0.0168–0.0437)	< 0.0001
EQ-VAS								
Q1	Ref.		Ref.		Ref.		Ref.	
Q2	3.1847 (1.5085–4.8609)	0.0002	2.8521 (1.1648–4.5393)	0.0009	2.8362 (1.1546–4.5179)	0.0009	1.3732 (–0.3484–3.0948)	0.1180
Q3	5.9959 (4.4052–7.5867)	< 0.0001	4.9901 (3.3333–6.6469)	< 0.0001	4.8969 (3.2409–6.5528)	< 0.0001	3.3038 (1.5753–5.0324)	0.0002
*12 months*								
EQ-5D-3L index								
Q1	Ref.		Ref.		Ref.		Ref.	
Q2	0.0351 (0.0206–0.0496)	< 0.0001	0.0291 (0.0146–0.0437)	< 0.0001	0.0301 (0.0156–0.0447)	< 0.0001	0.0137 (–0.0011–0.0286)	0.0691
Q3	0.0533 (0.0396–0.0670)	< 0.0001	0.0417 (0.0274–0.0560)	< 0.0001	0.0422 (0.0279–0.0566)	< 0.0001	0.0228 (0.0079–0.0377)	0.0027
EQ-VAS								
Q1	Ref.		Ref.		Ref.		Ref.	
Q2	1.9844 (0.3672–3.6017)	0.0162	1.6906 (0.0816–3.2995)	0.0395	1.7070 (0.0990–3.3150)	0.0375	1.0409 (–0.6224–2.7043)	0.2200
Q3	5.7644 (4.2296–7.2992)	< 0.0001	4.5214 (2.9415–6.1014)	< 0.0001	4.5360 (2.9526–6.1194)	< 0.0001	3.8877 (2.2170–5.5583)	< 0.0001

Ref: reference category; Set Q1 as the reference. EQ-5D-3L: 3-level EuroQol five dimensions; EQ-VAS: EQ visual analogue scale.

Note: Model 1 included sociodemographic variables (age, sex, educational level, household income, health insurance, and occupation class); model 2 included Model 1 plus medical history (hypertension, diabetes mellitus, hyperlipidaemia, and atrial fibrillation), current smoking status, and current drinking status; Model 3 additionally adjusted for physical activity, rehabilitation training status, and clinical scale scores (NIHSS, mRS, MoCA).

### Associations between gait speed levels and EQ-5D-3L dimensions

Subjects in the Q3 group were associated with lower odds of having problems with mobility (odds ratio [OR] 0.255 [95% CI 0.141–0.458, *p* < 0.0001]), self-care (OR 0.328, CI 0.167–0.646, *p* 0.0013), and usual activities (OR 0.353, CI 0.211–0.590, *p* < 0.0001) at the 3-month follow-up, after adjusting for all covariates, relative to the Q1 group (Table IV). However, there was no correlation between the gait speed group and 2 categories of the EQ-5D-3L (pain/discomfort and anxiety/depression) at 3 months after adjusting for confounding factors (Table IV).

**Table IV:** Association between acute-phase gait speed and having problem in each EQ-5D-3L dimension at 3 and 12 months post-stroke

EQ-5D-3L dimensions^a^	Unadjusted		Model 1		Model 2		Model 3	
	OR (95% CI)	*p*-value	OR (95% CI)	*p*-value	OR (95% CI)	*p*-value	OR (95% CI)	*p*-value
*3 months*								
Mobility								
Q1	Ref.		Ref.		Ref.		Ref.	
Q2	0.339 (0.224–0.513)	< 0.0001	0.370 (0.241–0.567)	< 0.0001	0.363 (0.236–0.559)	< 0.0001	0.518 (0.325–0.827)	0.0058
Q3	0.126 (0.074–0.215)	< 0.0001	0.149 (0.086–0.260)	< 0.0001	0.148 (0.085–0.258)	< 0.0001	0.255 (0.141–0.458)	< 0.0001
Self-care								
Q1	Ref.		Ref.		Ref.		Ref.	
Q2	0.265 (0.158–0.444)	< 0.0001	0.287 (0.168–0.490)	< 0.0001	0.284 (0.166–0.486)	< 0.0001	0.448 (0.251–0.797)	0.0063
Q3	0.135 (0.074–0.248)	< 0.0001	0.176 (0.094–0.331)	< 0.0001	0.177 (0.094–0.334)	< 0.0001	0.328 (0.167–0.646)	0.0013
Usual activities								
Q1	Ref.		Ref.		Ref.		Ref.	
Q2	0.427 (0.294–0.619)	< 0.0001	0.480 (0.326–0.707)	0.0002	0.465 (0.314–0.690)	0.0001	0.700 (0.456–1.073)	0.1017
Q3	0.167 (0.106–0.264)	< 0.0001	0.211 (0.131–0.340)	< 0.0001	0.207 (0.128–0.336)	< 0.0001	0.353 (0.211–0.590)	< 0.0001
Pain/discomfort								
Q1	Ref.		Ref.		Ref.		Ref.	
Q2	0.526 (0.350–0.789)	0.0019	0.527 (0.345–0.804)	0.0030	0.520 (0.339–0.797)	0.0027	0.695 (0.441–1.095)	0.1163
Q3	0.503 (0.343–0.739)	0.0005	0.514 (0.339–0.778)	0.0017	0.510 (0.336–0.775)	0.0016	0.738 (0.468–1.163)	0.1900
Anxiety/depression								
Q1	Ref.		Ref.		Ref.		Ref.	
Q2	0.833 (0.566–1.226)	0.3540	0.889 (0.594–1.330)	0.5670	0.893 (0.596–1.340)	0.5859	1.268 (0.811–1.983)	0.2982
Q3	0.560 (0.376–0.833)	0.0042	0.595 (0.388–0.912)	0.0171	0.594 (0.387–0.912)	0.0173	0.874 (0.542–1.409)	0.5797
*12 months*								
Mobility								
Q1	Ref.		Ref.		Ref.		Ref.	
Q2	0.517 (0.353–0.759)	0.0007	0.637 (0.425–0.955)	0.0289	0.612 (0.407–0.921)	0.0187	0.833 (0.536–1.296)	0.4179
Q3	0.225 (0.143–0.354)	< 0.0001	0.340 (0.210–0.550)	< 0.0001	0.332 (0.205–0.539)	< 0.0001	0.485 (0.289–0.812)	0.0060
Self-care								
Q1	Ref.		Ref.		Ref.		Ref.	
Q2	0.402 (0.247–0.654)	0.0002	0.453 (0.273–0.750)	0.0021	0.427 (0.256–0.713)	0.0011	0.629 (0.361–1.095)	0.1014
Q3	0.201 (0.114–0.354)	< 0.0001	0.263 (0.146–0.477)	< 0.0001	0.254 (0.139–0.463)	< 0.0001	0.412 (0.217–0.784)	0.0069
Usual activities								
Q1	Ref.		Ref.		Ref.		Ref.	
Q2	0.502 (0.349–0.723)	0.0002	0.547 (0.374–0.799)	0.0018	0.530 (0.361–0.780)	0.0013	0.703 (0.465–1.062)	0.0943
Q3	0.198 (0.128–0.308)	< 0.0001	0.243 (0.153–0.386)	< 0.0001	0.237 (0.149–0.378)	< 0.0001	0.325 (0.198–0.536)	< 0.0001
Pain/discomfort								
Q1	Ref.		Ref.		Ref.		Ref.	
Q2	0.575 (0.380–0.871)	0.0090	0.631 (0.410–0.969)	0.0355	0.622 (0.404–0.958)	0.0313	0.771 (0.484–1.228)	0.2739
Q3	0.358 (0.230–0.556)	< 0.0001	0.425 (0.266–0.678)	0.0003	0.426 (0.266–0.682)	0.0004	0.558 (0.335–0.930)	0.0252
Anxiety/depression								
Q1	Ref.		Ref.		Ref.		Ref.	
Q2	0.778 (0.516–1.171)	0.2288	0.894 (0.581–1.376)	0.6100	0.892 (0.578–1.376)	0.6042	1.187 (0.740–1.905)	0.4762
Q3	0.540 (0.355–0.821)	0.0040	0.686 (0.435–1.083)	0.1055	0.672 (0.425–1.064)	0.0900	0.906 (0.547–1.502)	0.7019

OR: odds ratio; 95% CI: 95% confidence interval; Ref: reference category, Set Q1 as the reference.

Note: Model 1 included sociodemographic variables (age, sex, educational level, household income, health insurance, and occupation class); Model 2 included Model 1 plus medical history (hypertension, diabetes mellitus, hyperlipidaemia, and atrial fibrillation), current smoking status, and current drinking status; Model 3 additionally adjusted for physical activity, rehabilitation training status, and clinical scale scores (NIHSS, mRS, MoCA).

a Defined G1 as the reference (G1: no problems; G2: some problems; G3: severe problems).

Compared with the Q1 group, patients in Q3 had a reduced risk of problems in 4 of the 5 of EQ-5D-3L, i.e., mobility, self-care, usual activities, and pain/discomfort at 12 months (Table IV). However, there was not a significant association between the gait speed group and anxiety/depression at 12 months after adjusting for confounding factors (Table IV).

## DISCUSSION

### Principal findings

The present study provided evidence for a significant association between acute-phase gait speed and quality of life after 3 and 12 months in patients with stroke. This study showed a positive correlation between gait speed and HRQoL at 3 and 12 months, even after controlling for multiple confounders. Participants in the highest tertile of gait speed levels tended to have higher EQ-5D-3L index and EQ-VAS scores. In the dimension-specific analysis, patients with faster gait speed were at significantly lower risk in 4 of the 5 dimensions of EQ-5D-3L, i.e., mobility, self-care, usual activities, and pain/discomfort at 12 months.

### Comparison with other studies and implications for research

The primary long-term rehabilitation objective for stroke survivors is to achieve higher HRQoL scores after acute clinical stabilization (23). Therefore, identifying variables that predict stroke-specific HRQoL can benefit the identification of rehabilitation strategies (24). The literature shows that acute-phase clinical measures can predict long-term functional outcomes after stroke (25). Both the NIHSS severity score and the level of disability assessed by mRS have been shown to be independent predictors of post-stroke HRQoL at 3 and 12 months (19, 24).

A recent study showed that extending early gait measures with HRQoL outcomes revealed that the 10MWT and the 6-Minute Walk Test (6MWT) in an inpatient rehabilitation facility can predict 3-month lower extremity HRQoL and function outcome after ischaemic stroke (26). Our results also confirm the idea that baseline gait speed could be predictive of HRQoL at 3 months post-stroke. In addition, our study found that gait speed at baseline was a predictor of HRQoL at 12 months post-stroke. The integration of multiple physiological systems, including the central and peripheral neurological systems, the perceptual system, the musculoskeletal system, and the energy production and/or delivery system, could be found in the higher-order function of gait (27). Gait speed is considered one of the most important physical traits and a sign of general health (28). Gait speed can be accurately measured in a few minutes by using only a 10-m walkway and a stopwatch, and it is inexpensive and easier to measure than other multiparametric and time-consuming devices for prognostic evaluation (11). Therefore, gait speed offers a global indicator of functional status and is less susceptible to subjectivity (27). An et al. (29) showed that gait speed is a better predictor than walking distance for community walking ability in moderately affected post-stroke survivors. Hong (30) showed that the Korean stroke population who performed community walking every day had higher HRQoL scores than the patients who had no walking or 1–3 walking days during a week. A study showed that gait speed is also associated with social participation, and patients with a faster gait may have more opportunities to engage in a variety of social activities (9). Therefore, gait speed is a good assessment indicator to predict future HRQoL. Thus it is important to improve quality of life at 3 and 12 months by improving gait speed in the acute phase.

In the dimension-specific analysis, there were distinct decreases in the chance of having problems in mobility, self-care, and usual activities in patients with stroke who had higher gait speed at 3 and 12 months. A cross-sectional study also showed that gait speed was related to the Stroke Impact Scale (SIS) score and its domains, such as strength, mobility, ability to perform activities of daily living (ADL), hand function, emotion, social participation, and thinking of stroke recovery (9). This finding can be explained in terms of the recovery course. At 3 months post-stroke, most stroke survivors suffered from some degree of physical dependence, and their capacity to move around and engage in daily activities was constrained. Patients continued to experience physical dysfunction even a year after the stroke. Regarding body functions, activities and participation were central factors in HRQoL at 3 months and may account for nearly all variation in HRQoL (31). However, this study found that the risk of having problems with pain/discomfort decreased in participants with higher gait speed at 12 months, while there was no significant association at 3 months. Previous studies have shown that about one-fifth of patients experience stroke-related pain at 1 year after first-ever stroke, and that stroke-related pain is associated with degree of hemiplegia at baseline and sensorimotor disorders in this patient group (32). The results of this study demonstrate that stroke patients with lower gait speed have limited mobility. A cross-sectional 5-year follow-up study showed that stroke survivors with restricted mobility had almost 4 times higher odds of experiencing more frequent pain (33). It may be speculated that stroke survivors with lower gait speed experience more pain at 1 year after stroke. However, the cause of the EQ-5D pain or discomfort dimension has not been determined but may be attributable to stroke conditions (e.g., headaches or joint stiffness), or non-stroke-related conditions (e.g., age-related changes such as arthritis or muscle soreness) (34). Additionally, HRQoL is a complex concept with several dimensions (35). Previous studies have shown that personality functions, energy and drive functions, gait patterns functions, and housework activities in the International Classification of Functioning, Disability and Health (ICF) framework are substantial factors in HRQoL at 1 year after stroke, explaining only half of the change in HRQoL (31). In particular, HRQoL was repeatedly influenced by temperament and personality functions, which are closely related to personal factors. Darlington et al. (36) also showed that coping strategies were an important determinant of HRQoL later in the chronic phase. Therefore, HRQoL is influenced not only by objective disability but also by changes in environmental and personal factors and expectations of quality of life (35). Thus, the determinants of HRQoL may be more ambiguous at 1 year after stroke (31). This is an area that should be explored in future research. In terms of clinical implications, an initial focus on patients’ walking abilities may have a significant impact on HRQoL.

### Strengths and limitations

This study has some strengths. The combination of long-term follow-up data in a large prospective stroke cohort examined the relationship between baseline gait speed and HRQoL at 12 months in patients with stroke. The association was evaluated by multivariable linear regression analysis after adjusting for various confounding factors. Moreover, we analysed the association between baseline gait speed and HRQoL in each of the 5 dimensions of the EQ-5D-3L at 3 and 12 months post-stroke.

Our study had several limitations. First, our sample included only those patients who were able to complete the 10MWT. Therefore, our study subjects were mainly those with only mild stroke (low NIHSS score) and the findings may have limited applicability to other stroke survivors. Second, we had no way of documenting the improvement in gait speed by patients completing rehabilitation and, therefore, were unable to assess the effects of changes in gait speed on HRQoL during the longitudinal study. Considering the predictive value of gait speed, future studies should assess the effect of the utility of interventions to improve gait speed on HRQoL. Third, the EQ-5D-3L instrument is a frequently used generic preference-based tool with only 3 descriptive levels for each of the 5 dimensions (37). Therefore, the information gained might be limited. Future studies using more sensitive disease-specific instruments are therefore warranted, and the EQ-5D-5 level scale (EQ-5D-5L) has obvious advantages that can reduce the ceiling effect compared with the EQ-5D-3L tool (38, 39).

### Conclusion

In this study, acute-phase gait speed could be predictive of post-stroke HRQoL at 3 and 12 months, especially when associated with more questions in specific domains of the EQ-5D-3L. Further prospective studies on improving HRQoL by increasing the acute-phase gait speed are needed.

## Supplementary Material

GAIT SPEED AT THE ACUTE PHASE PREDICTED HEALTH-RELATED QUALITY OF LIFE AT 3 AND 12 MONTHS AFTER STROKE: A PROSPECTIVE COHORT STUDY
