# Supplementary material for: GAIT SPEED AT THE ACUTE PHASE PREDICTED HEALTH-RELATED QUALITY OF LIFE AT 3 AND 12 MONTHS AFTER STROKE: A PROSPECTIVE COHORT STUDY
Source: J Rehabil Med. 2024 Apr 15;56:24102. doi: 10.2340/jrm.v56.24102 (PMC11031874; doi:10.2340/jrm.v56.24102)
Supplement: GAIT SPEED AT THE ACUTE PHASE PREDICTED HEALTH-RELATED QUALITY OF LIFE AT 3 AND 12 MONTHS AFTER STROKE: A PROSPECTIVE COHORT STUDY [file JRM-56-24102-s1.pdf]

Supplementary material has been published as submitted. It has not been copyedited, or typeset by Journal of Rehabilitation Medicine

Table SI. Sensitivity analysis of the EQ-5D-3L scores by using the UK value sets at 3 and 12 months based on gait speed

**Variable**	Total	Gait speed			*p*
		Q1 (≤0.80 m/s)	Q2 (0.80-1.10 m/s)	Q3 (≥1.10 m/s)	
Participants, n (%)	1,475	463 (33.0)	451 (30.9)	561 (36.1)	
**EQ-5D-3L index, mean (SD)**					
3 months	0.87 (0.12)	0.83 (0.16)	0.88 (0.12)	0.89 (0.08)	<0.0001
12 months	0.86 (0.14)	0.83 (0.18)	0.87 (0.13)	0.88 (0.12)	<0.0001

EQ-5D-3L: three-level EuroQol Five Dimensions; SD: standard deviation.

Table SII. Sensitivity analysis of the association between baseline gait speed and HRQoL score 3 and 12 months of follow-up

Outcomes	Gait speed	Unadjusted model		Adjusted model	
		B[a] (95%CI)	*P-value*	B[a] (95%CI)	*P-value*
3 months					
**EQ-5D-3L index**	Q1	Reference		Reference	
	Q2	0.0425(0.0266-0.0583)	<0.0001	0.0222(0.0059-0.0385)	0.0075
	Q3	0.0589(0.0439-0.0740)	<0.0001	0.0083(0.0154-0.0481)	0.0001
12 months					
**EQ-5D-3L index**	Q1	Reference		Reference	
	Q2	0.0392(0.0207-0.0577)	<0.0001	0.0177(-0.0016-0.0370)	0.0725
	Q3	0.0505(0.0329-0.0681)	<0.0001	0.0203(0.0009-0.0396)	0.0405

Set Q1 as the reference. All models included sociodemographic variables (age, sex, educational level, household income, health insurance, and occupation class)，medical history (hypertension, diabetes mellitus, hyperlipidemia, atrial fibrillation, and coronary heart disease), intravenous thrombolysis, current smoking, and current drinking, physical activity, rehabilitation training status (***3 months***: hospitalization and 3-months after discharge; ***12 months:*** hospitalization、3-months after discharge and 12-months after discharge), and clinical scale score (NIHSS, mRS, MoCA).
